# FaMYB5 Interacts with FaBBX24 to Regulate Anthocyanin and Proanthocyanidin Biosynthesis in Strawberry (*Fragaria* × *ananassa*)

**DOI:** 10.3390/ijms241512185

**Published:** 2023-07-29

**Authors:** Lianxi Zhang, Yiping Wang, Maolan Yue, Leiyu Jiang, Nating Zhang, Ya Luo, Qing Chen, Yong Zhang, Yan Wang, Mengyao Li, Yunting Zhang, Yuanxiu Lin, Haoru Tang

**Affiliations:** College of Horticulture, Sichuan Agricultural University, Chengdu 611130, China; zlx981027@163.com (L.Z.); wangyp_sicau@163.com (Y.W.); maolanyue@outlook.com (M.Y.); jiangleiyusicau@outlook.com (L.J.); zhangnating714@163.com (N.Z.); luoya945@sicau.edu.cn (Y.L.); supnovel@sicau.edu.cn (Q.C.); zhyong@sicau.edu.cn (Y.Z.); wangyanwxy@163.com (Y.W.); limy@sicau.edu.cn (M.L.); asyunting@sicau.edu.cn (Y.Z.); linyx@sicau.edu.cn (Y.L.)

**Keywords:** strawberry, transcription factor, anthocyanin, proanthocyanidin

## Abstract

MYB and BBX transcription factors play important roles in flavonoid biosynthesis. Here, we obtained transgenic woodland strawberry with stable overexpression of *FaMYB5*, demonstrating that FaMYB5 can increase anthocyanin and proanthocyanidin content in roots, stems and leaves of woodland strawberry. In addition, bimolecular fluorescence complementation assays and yeast two-hybridization demonstrated that the N-terminal (1-99aa) of FaBBX24 interacts with FaMYB5. Transient co-expression of *FaBBX24* and *FaMYB5* in cultivated strawberry ‘Xiaobai’ showed that co-expression strongly promoted the expression of *F3′H*, *4CL-2*, *TT12*, *AHA10* and *ANR* and then increased the content of anthocyanin and proanthocyanidin in strawberry fruits. We also determined that FaBBX24 is also a positive regulator of anthocyanin and proanthocyanidin biosynthesis in strawberry. The results reveal a novel mechanism by which the FaMYB5–FaBBX24 module collaboratively regulates anthocyanin and proanthocyanidin in strawberry fruit.

## 1. Introduction

Strawberry (*Fragaria* × *ananassa* Duch.) is full of macronutrients and bioactive ingredients, such as vitamins, minerals and flavonoids. It has high nutritional and healthcare value, especially its health effect of preventing cardiovascular disease, cancer and other diseases [1], which makes it popular with consumers. The flesh of the strawberry is actually enlarged from the receptacle, and the real fruit (achene) is the seed-like object on the surface, so we often say that the strawberry fruit actually refers to the edible fleshy part of the strawberry. Flavonoid compounds are one of the important secondary metabolites of plants and are widely distributed in the plantae. Because of flavonoids’ strong biological activity, they often act as an antioxidant or signaling molecule to regulate cell function [2]. At present, there have been extensive studies on flavonoid synthesis pathways in horticultural crops [3,4]. There are two main types of genes involved in flavonoid metabolism. One is structural genes that are involved in flavonoid synthesis by directly encoding various biosynthetic enzymes. These include *PAL* (phenylalanine lyase), *4CL* (4-cinnamate-CoA ligase), *CHS* (Chalcone synthase), *CHI* (Chalcone isomerase), *F3H* (flavanone 3-hydroxylase), *F3′H* (flavonoid 3′-hydroxylase), *DFR* (dihydroflavonol 4-reductase), *ANS* (anthocyanin synthase), *ANR* (Anthocyanidin reductase), *LAR* (leucoanthocyanidin reductase) and *UFGT* (UDP-glucose: flavonoid 3-o-glucosyltransferase) [5], some genes that control transport and modify enzymes such as *TT10* (laccase 15), *TT12* (MATE transporter), *TT19* (glutathione-S-transferase) and *AHA10* (H^+^-ATPase) [6]. The other type of gene is transcription factors (TFs) which regulate flavonoid metabolism by regulating structural genes [7].

V-myb myeloblastosis viral oncogene homolog (MYB) proteins are one of the largest families of plant transcription factors [8]. MYB transcription factors have regulatory effects on metabolic pathways such as anthocyanin and proanthocyanidin in flavonoids and can affect flower organ development and fruit coloring in plants [9]. Anthocyanin synthesis in plants is mainly regulated by the transcription complex (MBW complex) composed of MYB, basic helical loop helix (bHLH) and WD-repeat (WDR) proteins [10]. Since the discovery of ZmC1 [11], the first MYB transcription factor regulating anthocyanin in maize, regulation of anthocyanin by MYB transcription factors has been reported in many plants. The expression level of anthocyanin biosynthesis structural gene in transgenic *Arabidopsis thaliana* overexpressed *AgMYB2*, which was significantly up-regulated [12]; carrot DcMYB6 can directly activate *DcUCGXT1* and *DcSAT1* to regulate the glycosylation and acylation of anthocyanin [13]. Apple MdMYBA specifically bound to the *MdANS* promoter, increasing anthocyanin accumulation in apple [14]; MdMYB3, MdMYB9, MdMYB10 and MdMYB114 can promote apple fruit coloring by interacting with bHLH3 and WD40 [15,16,17]; MdMYB306 interacted with MdbHLH33 to regulate structural genes *F3H*, *DFR* and *UFGT* to regulate anthocyanin synthesis [18]. The complex formed by pear PyMYB10, PybHLH and PyWD40 transcription factors positively regulates pear anthocyanin synthesis [19]. Peach PpMYB10.1 and PpMYB10.3 interacted with PpbHLH proteins to activate the expression of *CHS*, *F3′H* and *UFGT*, thereby promoting the synthesis of anthocyanin [20]. In strawberry, FaMYB5/FaMYB10-FaEGL3 (bHLH)-FaLWD1/FaLWD1-like (WD40) formed an ‘MBW’ complex to positively regulate anthocyanin synthesis in strawberry fruit [21,22], FaMYB10 promotes anthocyanin synthesis in strawberry, and *FaMYB10* mutation is the main cause of color difference between yellow woodland strawberry ‘Yellow wonder’ and red woodland strawberry ‘Ruegen’ [23,24]. The interaction between FaMYB123 and FabHLH3 in strawberry regulates anthocyanin synthesis by regulating late biosynthetic genes (LBGs) [25]. FaMYB1 inhibited anthocyanin synthesis, while FaMYB9 and FaMYB11 promoted proanthocyanidin synthesis [26].

The B-BOX protein family plays an important role in plant photomorphogenesis [27,28], the flowering cycle [29,30] and stress of adversity [31,32,33]. In recent years, many studies on the regulation of plant anthocyanin metabolism by B-BOX proteins have been reported. Strawberry FaBBX22 can promote the expression of anthocyanin structural genes, and the interaction between FaHY5 and FaBBX22 can enhance this effect [34]. In apple, MdBBX1 regulated anthocyanin accumulation by regulating *MdMYB10* and *DFR* [35], and MdBBX21 interacted with MdHY5 to promote *MdMYB1* and thus regulated anthocyanin synthesis [36]. PpBBX18 formed a heterodimer with PpHY5 to regulate anthocyanin accumulation in pear fruit [37]. Overexpression of *PpBBX16* promoted anthocyanin accumulation in pear callus and was a positive regulator of photoinduced anthocyanin accumulation, and the presence of PpHY5 was required to activate its full function [38]; overexpression of *VvBBX44* inhibited *UFGT* expression and anthocyanin accumulation in grapes callus [39]; and PavBBX6 and PavBBX9 positively regulate light-induced anthocyanin and ABA biosynthesis by promoting *PavUFGT* and *PavNCED1* expression in sweet cherry [40]. Overexpression of *PtrBBX23* in poplar activated expression of MYB TFs and structural genes in the flavonoid pathway, thereby promoting the accumulation of proanthocyanidin and anthocyanin in poplar [41]. Although the functions of some transcription factors have been clearly defined, their interaction networks and mechanisms remain unclear.

In previous studies, we evaluated FaMYB5 as playing an important role in anthocyanin and proanthocyanidin metabolism through transcriptomics and metabolomics [21,22]. In this study, FaMYB5 was stably overexpressed in woodland strawberry to confirm that FaMYB5 can promote anthocyanin and proanthocyanidin accumulation in strawberry plants. We found that FaBBX24, a member of the strawberry BBX family, interacts with FaMYB5 to synergistically regulate anthocyanin accumulation. The aim of this study is to improve the flavonoid metabolic network regulated by FaMYB5 and enrich the understanding of the flavonoid metabolic regulatory network in strawberry.

## 2. Results

### 2.1. Overexpression of FaMYB5 Promoted the Accumulation of Anthocyanin and Proanthocyanidin in Woodland Strawberry

**Figure 1:**
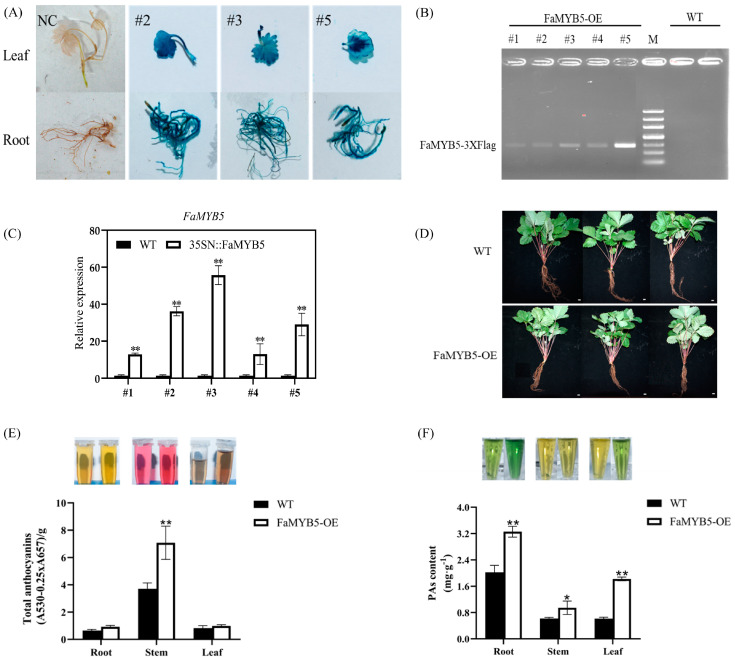
Transgenic woodland strawberry with stable overexpression of *FaMYB5*. (**A**) *FaMYB5* was expressed in roots, stems and leaves with GUS staining; roots and leaves of wild-type woodland strawberries, which could not be stained by GUS, were used as negative controls. (**B**) Compared with the wild-type, the transgenic plants could amplify the special bands of FaMYB5-3XFLag. (**C**) The relative expression levels of *FaMYB5* in transgenic lines using RT–qPCR. (**D**) The wild-type and FaMYB5-overexpressed plants with the same growth status were selected to determine the content of anthocyanin and proanthocyanidin in leaves, stems and roots; with three biological replicates, bar = 10 cm. (**E**,**F**) Overexpression of FaMYB5 significantly increased the anthocyanin content in stems and proanthocyanidin in roots, stems and leaves compared with wild-type. The red color of the solution indicates the content of anthocyanins; blue solution indicates the content of proanthocyanidin. The proanthocyanidin solution was diluted 20× at the time of determination, and the color was light. Error bars are SEs for three replicates. Significant differences with wild-type were compared using Student’s *t* test (** *p*  < 0.01; * *p* <  0.05).

In our previous studies, transient overexpression of cultivated strawberries showed that FaMYB5 positively regulates flavonoid metabolism in strawberry fruits [21]. To reveal the regulatory role of FaMYB5 in the whole life cycle of strawberry, the pCAMBIA1301-FaMYB5-3XFlag vector was constructed, and the cotyledon of diploid strawberry ‘Ruegen’ was infected with *Agrobacterium tumefaciens*. Three independent transgenic lines #2, #3 and #5, with high expression levels were identified using GUS staining (Figure 1A), PCR identification (Figure 1B) and RT–qPCR identification (Figure 1C) detection. Anthocyanin and proanthocyanidin in roots, stems and leaves of transgenic plants were measured, and wild-type woodland strawberry with similar growth was used as a control (Figure 1D). The results showed that overexpression of *FaMYB5* increased the anthocyanin content in stems (Figure 1E) and the proanthocyanidin content in roots, stems and leaves (Figure 1F) of woodland strawberry.

### 2.2. B-BOX Protein FaBBX24 Interacts with FaMYB5

**Figure 2:**
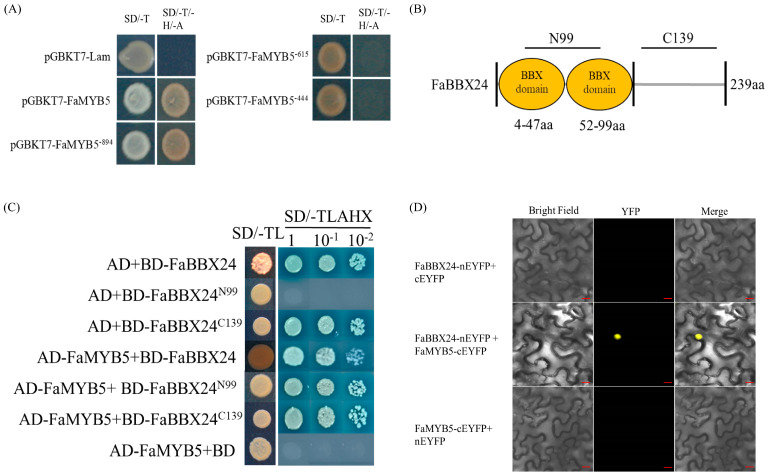
FaMYB5 interacts with FaBBX24. (**A**) FaMYB5 trans-acting activity verification. SD/-T, SD/-Trp medium, SD/-T/-H/-A, SD/-Trp/-His/-Ade medium. n yeast cell. The yeast cells transformed with pGBKT7-Lam vector were used as a negative control. (**B**) The 4-47aa of FaBBX24 belong to B-BOX domain 1, and the 52-99aa belong to B-BOX domain 2, both of which constitute the N terminus of FaBBX24. (**C**) BD-FaBBX24^N99^ interacted with AD-FaMYB5 using yeast two-hybrid, SD/-TL, SD/-Trp/-Leu medium, SD/−TLAHX, SD/-Trp/-Leu/-Ade/-His/X-α-gal. (**D**) Physical association between FaMYB5 and FaBBX24 confirmed with BiFC assay. FaBBX24-nEYFP/ FaMYB5-cEYFP co-transformed tobacco leaves, with FaBBX24-nEYFP/cEYFP and FaMYB5-cEYFP/nEYFP as negative controls; bars = 20 μm.

The yeast two-hybrid screening library was used to search for proteins that can interact with FaMYB5. Full-length FaMYB5 showed strong trans-acting activity; then, the *FaMYB5* sequence was cut and a longer fragment FaMYB5^−615^ was selected for further library screening (Figure 2A). A total of 189 potential interacting proteins were screened (Table S1). The potential protein FaBBX24 was inserted into pGBKT7, and BD-FaBBX24 has trans-acting activity. According to the B-BOX domains, FaBBX24 is divided into FaBBX24^N99^ and FaBBX24^C139^ for yeast two-hybrid (Figure 2B). The results show that BD-FaBBX24^N99^ interacts with AD-FaMYB5 (Figure 2C). BiFC assay also demonstrated that FaBBX24 interacts with FaMYB5 in vivo (Figure 2D).

### 2.3. Bioinformatics Analysis and Tissue Expression Pattern of FaBBX24

**Figure 3:**
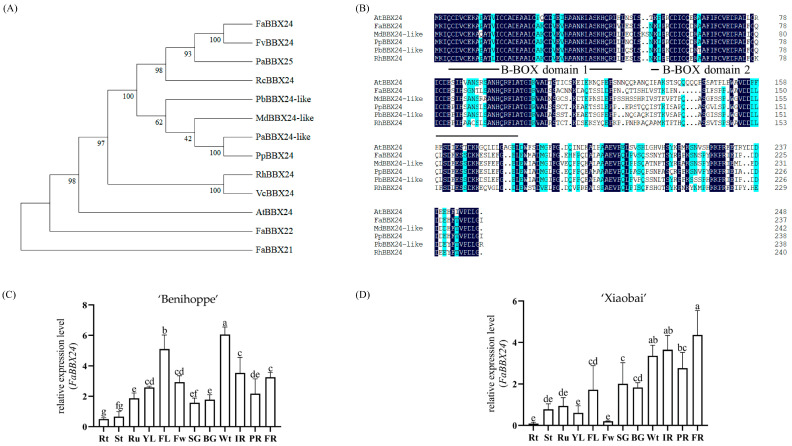
Bioinformatics analysis and spatiotemporal expression patterns in strawberry tissues of *FaBBX24*. (**A**) Phylogenetic analysis of FaBBX24 with its homologous proteins from other species. The phylogenetic tree was constructed using the Neighbor-Joining Tree Method in MEGA6, and the bootstrap was 1000 replicates. (**B**) Multiple protein sequence alignment of FaBBX24 with other BBX24 proteins. The black line marks the B-BOX domain. The gene accession numbers that have appeared above are as follows: FvBBX24(XM_004309761.2), PaBBX25(XM_050517417.1), RcBBX24(XM_024337817.2), PbBBX24-like(XM_048579829.1), MdBBX24-like(NM_001328919.1), PaBBX24-like(XM_021967653.1), PpBBX24(XM_007223749.2), RhBBX24(OL690502.1), VcBBX24(OP957064.1), LbBBX24(MH813941.1) and AtBBX24(NM_100484.4). (**C**,**D**) Expression pattern of *FaBBX24* in different tissues of cultivated strawberry ‘Benihoppe’ and ‘Xiaobai’. Rt—root; St—stem; Ru—runner; YL—young leaf; FL—functional leaf; Fw—flower; SG—small green; BG—big green; Wt—white; IR—initially red; PR—partially red; FR—full red. Error bars are SEs for three replicates. Different letters above the bars indicate significantly different values (*p* < 0.05) according to a Least Significant Difference (LSD) test.

FaBBX24 was cloned from cultivated strawberry ‘Benihoppe’. FaBBX24 contains a 717 bp open reading frame (ORF) encoding 238 amino acids, with a predicted protein size of 26.2 kDa and an average isoelectric point (PI) of 4.624. Phylogenetic tree analysis showed that FaBBX24 was closely related to FvBBX24 and clustered with RcBBX24 and PaBBX24 (Figure 3A). Multiple protein sequence alignment showed that FaBBX24 was similar to *Arabidopsis thaliana* AtBBX24, containing B-BOX domain 1 and B-BOX domain 2 and belonging to Group IV B-BOX proteins [42] (Figure 3B). The temporal and spatial expression patterns of *FaBBX24* in different tissues of strawberry ‘Benihoppe’ and ‘Xiaobai’ were determined with RT–qPCR (Figure 3C,D). The results showed that *FaBBX24* was expressed in both the fruit development stages and vegetative organs of cultivated strawberry ‘Benihoppe’, with the highest expression level in the white stage and functional leaf and the lowest expression level in the root and stem. In cultivated strawberry ‘Xiaobai’, the expression level of *FaBBX24* gradually increased with fruit ripening and reached the maximum in the full red stage, while the expression level was low in the root, stem, leaf and flower.

### 2.4. FaMYB5 Interacted with FaBBX24 to Promote Anthocyanin and Proanthocyanidin Accumulation in Strawberry Fruits

**Figure 4:**
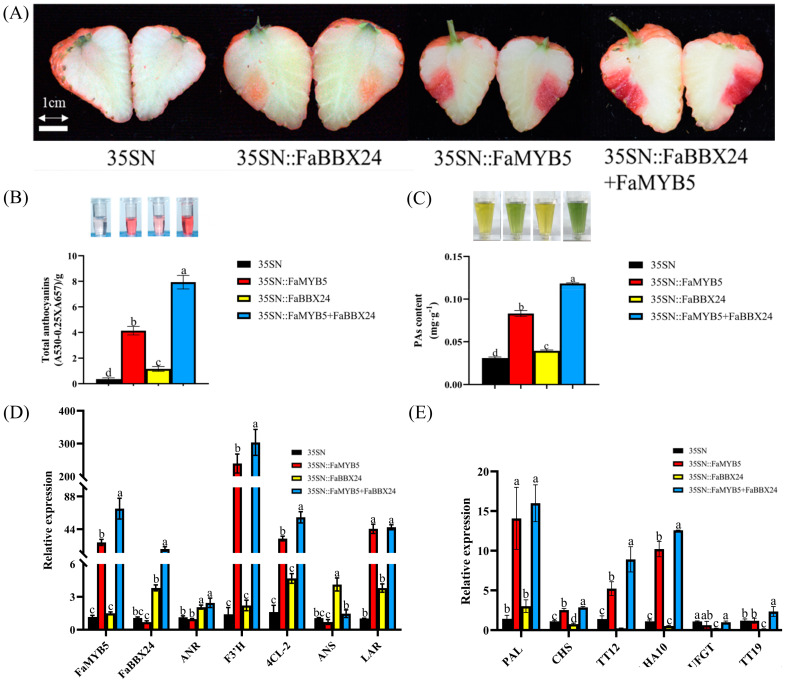
Co-expression of *FaMYB5* and *FaBBX24* in strawberry fruits. (**A**) Transient overexpression of *FaMYB5* and *FaBBX24* in cultivated strawberry ‘Xiaobai’. Bars = 1 cm. (**B**,**C**) Anthocyanin and proanthocyanidin content in strawberry fruits with overexpression of *FaBBX24* and *FaMYB5*. The red color of the solution indicates the content of anthocyanins; blue solution indicates the content of proanthocyanidin. (**D**,**E**) The relative expression levels of anthocyanin and proanthocyanidin structural genes in strawberry fruits overexpressing *FaMYB5* and *FaBBX24* were determined with RT–qPCR. Error bars are SEs for three replicates. Different letters above the bars indicate significantly different values (*p* < 0.05) compared to 35SN according to a Least Significant Difference (LSD) test.

In previous studies, we found that FaMYB5 can up-regulate structural genes *PAL*, *C4H*, *F3′H* and *LAR* and can directly regulate *F3′H* and *LAR* promoters to participate in anthocyanin and proanthocyanidin metabolism [21]. In this study, *FaMYB5*, *FaBBX24* and co-expression of *FaMYB5* and *FaBBX24* were transiently overexpressed in cultivated strawberry ‘Xiaobai’ (Figure 4A). Transient overexpression of *FaBBX24* was able to induce anthocyanin accumulation in strawberry fruits (Figure 4A). RT–qPCR results show that FaBBX24 was able to significantly up-regulate the anthocyanin structural gene *ANS* (Figure 4D). Co-expression of *FaMYB5* and *FaBBX24* significantly increased anthocyanin and proanthocyanidin content in fruit (Figure 4B,C); moreover, co-expression significantly increased the expression levels of structural genes *F3′H*, *4CL-2*, *TT12* and *AHA10* (Figure 4D,E).

## 3. Discussion

In previous studies, transient overexpression of *FaMYB5* in strawberry fruits has been shown to promote anthocyanin and proanthocyanidin accumulation in strawberry fruit. In this study, we obtained stable *FaMYB5* overexpression plants and measured the anthocyanin content in each organ. The results show that compared with wild-type, overexpression of *FaMYB5* promoted the accumulation of anthocyanin and proanthocyanidin in roots, stems and leaves, which may help plants resist adversity [43].

The MYB protein family plays an important role in plant phenylpropane metabolism [44]. According to the number and position of its domains, it has been divided into four subclasses: 1R (R1/R2, R3-MYB), 2R (R2R3-MYB), 3R (R1R2R3-MYB) and 4R (containing four similar R1/R2 repeats) [45], with the 2R protein being the largest class of MYB factors in plants. They are grouped into 22 subgroups on the basis of the conserved amino acid sequence motifs present in their most C-terminal MYB domain [46], which are involved in primary and secondary metabolism and determine cell life and properties, hormone signaling, developmental regulation and responses to biological and abiotic stresses [47,48,49]. In recent years, more and more studies on the regulation of flavonoid metabolism by R2R3-MYB have been reported, including studies on PtMYB6 [50], NtMYB330 [51], AN2like [52] and CaMYB39 [53]. The lack of R2 motif in FaMYB5 suggests that FaMYB5 is a potential negative regulator of anthocyanin synthesis [26]. In previous studies [21,22] and this study, we have demonstrated that FaMYB5 is a typical R2R3-MYB protein and positively regulates anthocyanin and proanthocyanidin biosynthesis in strawberry.

Flavonoids such as anthocyanin and proanthocyanidin are important products of phenylpropanoid metabolism. In addition to directly regulating structural genes of flavonoid biological metabolism, MYB proteins usually form complexes with other proteins to perform functions, such as the MYB-bHLH-WD40 complex [54,55]. It has also been reported that proteins other than bHLH protein interact with MYB transcription factors to regulate flavonoid metabolism. For example, Pp4ERF24 and Pp12ERF96 in pear were able to enhance the expression of *PpUFGT* and promoted anthocyanin synthesis through interaction with PpMYB114 [56], and PyERF3-PyMYB114-PybHLH3 complex promoted anthocyanin accumulation in pear peel by regulating target gene *ANS* [57]. Jasmonic acid domain protein interacted with AtMYB75 and bHLH protein TT8/GL3/EGL3, suggesting that it may promote anthocyanin synthesis and trichome initiation by regulating MBW complex and activating downstream signaling cascade [58]. The GARP-type transcription factor GLK1 regulated anthocyanin biosynthesis in *Arabidopsis thaliana* by interacting with MYB75, MYB90 and MYB113 [59]. COP1/SPA interacted with PAP1 and PAP2, which can affect their functions at the transcription and translation levels, and then regulated anthocyanin synthesis in *Arabidopsis thaliana* [60]. MYB75 interacted with MPK4, which regulates phosphorylation of MYB75 through photoinduction to maintain its stability, and played an important role in photoinduced anthocyanin synthesis in *Arabidopsis thaliana* [61]. In apples, MdBT2 interacted with MdMYB1 and target ubiquitination to degrade MdMYB1 and regulated the biological metabolism of anthocyanin [62]. MdNAC42-MdMYB10 was an important component of the regulatory network controlling anthocyanin accumulation in red-flesh apples [63]. MdbZIP44 and MdERF78 partnered with MdMYB1 to activate downstream target genes and promoted anthocyanin accumulation [64,65]. Here, we reveal the BBX protein and MYB protein interaction module and speculate that FaBBX24 interacted with FaMYB5 to enhance the transcriptional activation of FaMYB5 on *F3′H*, *TT12*, *AHA10* and *4CL-1* and promoted anthocyanin accumulation in strawberry fruits.

In this study, we screened the B-BOX protein FaBBX24, whose N-terminal contains two B-BOX domains that are involved in protein–protein interactions [66]. B-BOX proteins, as an important participant in the plant light-signaling pathway, have been shown to play an important role in light-mediated anthocyanin accumulation [67,68]. Among them, AtBBX20 [69], AtBBX21 [70], MdBBX20 [71], MdBBX21 [36], MdBBX22 [72], PpBBX16 [38], PpBBX18 [37], PavBBX6/9 [40] and FaBBX22 [34] have been proven to be positive regulators of anthocyanin, and AtBBX19 [73], AtBBX24 [74], AtBBX25 [75], AtBBX32 [76], PpBBX21 [77], PpBBX24 [78], MdBBX37 [79], MdCOL4 [80] and VvBBX44 inhibit anthocyanin biosynthesis. The same transcription factor in different species may perform opposite functions. MdMYB1 plays a central role in promoting anthocyanin accumulation and directly regulates anthocyanin structural genes [81,82], while MYB1 in strawberry is a repressor of flavonoid metabolism [26,83,84]. It has been reported that PpBBX24 causes the red skin of ‘Zaosured’ due to the deletion of 14 nucleotides [78], and these results indicated that PpBBX24 negatively regulated anthocyanin in pear. The amino acid sequence of FaBBX24 and PpBBX24 is highly similar (80.91%), but this study showed that FaBBX24 plays a role in promoting anthocyanin accumulation in strawberry fruits. Previous studies have found that FaMYB5 can promote the biosynthesis of proanthocyanidin in strawberry fruits by combining with *LAR* promoter. This research proves that the interaction between FaMYB5 and FaBBX24 did not further increase the *LAR* expression level. It is speculated that the increase in proanthocyanidin content after co-expression was due to the regulation of proanthocyanidin structural gene *ANR* by FaBBX24. Therefore, FaBBX24 is a positive regulator of anthocyanin and proanthocyanidin biosynthesis in strawberry fruits.

B-BOX proteins usually interact with HY5 and act as upstream MYB transcription factors such as MYB1 or MYB10 to regulate anthocyanin biosynthesis [28,79]. FaMYB5 has been proven to be a key transcription factor for anthocyanin metabolism in strawberry. However, RT–qPCR data showed that the expression level of *FaMYB5* did not change in strawberry fruits with *FaBBX24* overexpression. Therefore, whether FaBBX24 can directly regulate FaMYB5 remains to be further explored.

## 4. Materials and Methods

### 4.1. Plant Materials and Growth Conditions

The plant materials were woodland strawberry (*Fragaria vesca*) ‘Ruegen’ and its aseptic tissue culture seedlings; tobacco (*Nicotiana benthamiana*), which was grown in a greenhouse of the Horticulture College of Sichuan Agricultural University; and the cultivated strawberry (*Fragaria × ananassa*) ‘Benihoppe’ and ‘Xiaobai’ fruits which were picked from Xin Yue Strawberry Garden (Chengdu, China). Environmental conditions were controlled at 22 ± 2 °C, 80–90% relative humidity and 16/8 h light–dark cycle with 220 μmol m^−2^ s^−1^. The developmental stages of fruit are defined as small green, big green, white, partially red and full red [21]. All collected samples were immediately snap-frozen with liquid nitrogen and stored at −80 °C for further use.

### 4.2. Gene Clone and RT–qPCR Analysis

The total RNA of fruit samples was extracted using the improved CTAB method [85] and then reversed using RT Easy^TM^ II (with gDNase) (Foregene, Chengdu, China). cDNA was diluted tenfold and used as a template for gene cloning. The FaBBX24 sequences are shown in Figure S1. Cloning primers were designed using SnapGene software, as shown in Table S2 (all primers used in this article are in this table).

RT–qPCR was performed by a CFX Connect real-time system ((Bio-rad, Hercules, CA, USA), and the interspace 26S-18S RNA was used as a reference gene [86]. Primers were designed in NCBI (https://www.ncbi.nlm.nih.gov/) [Accessed on 20 April 2020], and the relative expression level was analyzed using the 2^−ΔΔCt^ method [87]. Hieff^®^ qPCR SYBR Green Master Mix (No Rox) (Yeasen Biotechnology Co., Ltd., Shanghai, China) was used for detecting the PCR products on a CFX96 Real-time reaction system (Bio-Rad, Hercules, CA, USA). Three biological replicates were set, and each biological replicate was set with three technical replicates.

### 4.3. Strawberry Genetic Transformation

FaMYB5 coding sequence (previously cloned in the laboratory) was inserted into pCAMBIA1301-Chip (with 3X Flag tag), and strawberry leaf rounds were infected with *Agrobacterium*-mediated genetic transformation. The specific method is as follows: strawberry leaf rounds were infected with *Agrobacterium tumefaciens* GV3101 containing pCAMBIA1301-FaMYB5-Chip (with 3X Flag tag); then, the leaf rounds were placed in 25 mL medium (MS salts + B5 vitamins +6-BA 3.0 mg·L^−1^ + IBA 0.2 mg·L^−1^ + 2% Sucrose + 1% Glucose + 0.25% Phytagel + 100 μM Acetosyringone, pH 5.5) in a petri dish for dark culture at 25 °C for 2 days. The leaf rounds were transferred to fresh culture bottles (MS salts + B5 vitamins + 6-BA 3.0 mg/L + IBA 0.2 mg/L + 3% Sucrose + 0.25% Phytagel + 250 mg/L Timentin + 250 mg/L Carbenicillin + 5 mg/L Hygromycin B, pH 5.8), 22 °C, 16 h (light) /8 h (dark), light intensity of 40 μmol·m^−2^·s^−1^ (about 2200 lx) for 15 days. The culture medium was then transferred every 20 days, during which the necrotic tissue was carefully excised and part of the green callus was transferred to a new screening medium until adventitious buds developed. When the indeterminate bud grew to 0.5–1 cm, it was removed from the base and transferred to a secondary medium (MS + 6-BA 0.2 mg/L + GA3 0.2 mg/L + 5 mg/L Hygromycin B + 3% Sucrose + 0.7% Agar, pH 5.8) for further culture. After identification of positive plants, 2–3 cm adventitive buds were cut off and transferred to rooting medium (1/2 MS +5 mg/L Hygromycin B + 3% Sucrose + 0.7% Agar, pH 5.8) under the same culture conditions as above to obtain intact plants.

### 4.4. Yeast Two-Hybrid Assay

The yeast two-hybrid assays were performed, according to the manufacturer’s instructions using the Matchmaker Gold Yeast Two-Hybrid System kit (Takara, Beijing, China). The coding sequence encoding the N-terminal of FaBBX24 (amino acids 1–99) and the C-terminal of FaBBX24 (amino acids 100–238) was cloned into pGBKT7 (Clontech) to generate the bait vector (BD-FaBBX24^N99^, BD-FaBBX24^C139^) containing the GAL4 DNA-BD sequence. The full-length coding sequence of FaMYB5 was individually cloned into pGADT7 vector (Clontech) to produce the prey vectors (AD-FaMYB5) containing the sequence encoding the GAL4 activation domain (AD). Yeast strain AH109 was co-transformed with the bait and prey vectors, and then protein interactions were evaluated on the basis of the ability of the cells to grow on synthetic defined (SD) medium lacking Leu, Trp, His, Ade and +X-α-gal after 4 days of growth at 28 °C.

### 4.5. BiFC Assay

The candidate genes were amplified with PCR using primers with restriction sites, and the fluorescent complementary vectors pXY104-cEYFP-FaMYB5 and pXY103-nEYFP-FaBBX24 were constructed. The recombinant plasmids were transformed into *Agrobacterium tumefaciens* GV3101, and the transformed positive and negative plasmids were used as control plasmids and then injected into tobacco leaves. Next, 48 h later, Laser Scanning Confocal Microscope (FV3000, OLYMPUS, Tokyo, Japan) was used to observe fluorescence signals in tobacco cells.

### 4.6. Transient Overexpression in Strawberry Fruit

The CDS of *FaBBX24* and *FaMYB5* were inserted into pCAMBIA1301 vector and transferred into *Agrobacterium tumefaciens* GV3101. A 1 mL sterile microsyringe was used to inject 300–500 μL subcutaneously into the fruit pedicles of strawberries at white stage. The strawberries after injection were dark cultured overnight at 18 °C and then transferred to normal material culture conditions; the steps of transfection are described in detail elsewhere [88].

### 4.7. Anthocyanin and Proanthocyanidin Content Measurement

The measurement of anthocyanin has been reported previously [89]. Specifically, 0.2 g plant material (extract: fresh weight of plant = 10:1) was ground with liquid nitrogen, and 2 mL anthocyanin extract (methanol:H_2_O:formic acid:trifluoroacetic acid, 70:27:2:1) was placed in a 40 °C water bath to avoid light for 4 h, followed by centrifugal collection of supernatants with UV spectrophotometer (UV-530pc, MAPADA, Shanghai, China) to measure the absorbance of A530, A657. The calculation formula is total anthocyanin = [A530 − (0.25 × A657)]/M, where A530 and A657 are absorbance at corresponding wavelengths, and M is fresh weight of plants.

Proanthocyanidin content measurement was described previously [90]. Specifically, 0.15 g of the sample was weighed (fully ground with liquid nitrogen), and 3 mL of proanthocyanidin extract (acetone:water:glacial acetic acid = 150:49:1) was added and shaken at 150 rpm for 1 h at 20 °C. The samples were centrifuged at 12 °C for 20 min at 10,000 rpm, and the supernatant was retained as the proanthocyanidin extract. After the extraction solution, 80% ethanol and 1% DMACA solution were mixed at 1:9:30, substance. Varioskan™ LUX (Thermo Fisher Scientific, Waltham, MA, USA) was used to determine OD value at 640 nm. Proanthocyanidin B2 is the standard.

### 4.8. Statistical Analysis

All the experimental data are expressed as the mean ± standard deviation from the mean (SD). The statistical analysis was performed using Student’s *t* test (** *p* < 0.01) and Least Significant Difference (LSD) test in IBM SPSS Statistics software, version 28.0 (IBM, Chicago, IL, USA).

## 5. Conclusions

In summary, by obtaining *FaMYB5* overexpression plants and transient overexpression of *FaMYB5* and *FaBBX24*, we determined that FaMYB5 and FaBBX24 are promoting factors for anthocyanin and proanthocyanidin biosynthesis of strawberry. It was also proven that FaMYB5 can further promote the expression of anthocyanin and proanthocyanidin structural genes *F3′H*, *TT12*, *AHA10*, *4CL-2* and *ANR* by forming a complex with FaBBX24 and can regulate the biosynthesis of anthocyanidin and proanthocyanidin in strawberry fruit. This study provides new insight into the transcriptional regulation of anthocyanin and proanthocyanidin by FaMYB5 and FaBBX24 in strawberry.

## Data Availability

Not applicable.

## Supplementary Materials

The following supporting information can be downloaded at: https://www.mdpi.com/article/10.3390/ijms241512185/s1.
