# Supplementary material for: FaMYB5 Interacts with FaBBX24 to Regulate Anthocyanin and Proanthocyanidin Biosynthesis in Strawberry (Fragaria × ananassa)
Source: Int J Mol Sci. 2023 Jul 29;24(15):12185. doi: 10.3390/ijms241512185 (PMC10418308; doi:10.3390/ijms241512185)
Supplement: Supplementary file 1 [file ijms-24-12185-s001.zip › Supplementary Materials/Figure S1.pdf]

The coding sequences of FaBBX24

>FaBBX24

ATGAAAATTCAGTGCGATGTGTGTGAGAAAGCTCCGGCGACGGTGATCTGTTGCGCTG
ACGAGGCGGCCCTCTGTGCCAAATGCGATGTGGAAGTGCACGCAGCAAACAAACTCG
CCAGCAAGCATCAGAGGCTTGTTCTTGAATCTCTCTCCAACAAGCTCCCTAGATGTGAT
ATATGCCAAGATAAGGCTGCTTTTATCTTCTGCGTTGAAGACAGAGCCCTGTTTTGTCA
GGACTGTGATGAATCAATTCATTCAGGAAATACCCTCTCTGCCAACCACCAGAGGTTCC
TCGCCACCGGAATCCGCGTTGCTTTGAGCTCCGCCTGTAATAACAAGGACGCTCAAAC
AAGTAGCTTAGACCCACCCAATCAAACTACTTCACACCTAGTTTCAACCAAACTGCCTA
ATTCTCTATTCTCGCCTCCTTGGGCGGTTGATGACTTGCTCCAGTTATCAGATTTTGAAT
CTTCTGACAAGAAAGAGTCGCTTGAGTTTGGAGAGCTTGAATGGATGGCAGATATGGG
TCTTTTTGGTGAGCATTTTCCTCAGGATGCTTTGGCAGCAGCTGAAGTACCTCAGCTTC
CAGTATCACAATCTAGCAACTATACATCATACAGACCCCCGAAATCAAACAGTCCCTAC
AAGAAGCCTAGGATTGAAATCCCGGATGATGATGATGAGCATTTCACTGTTCCTGATCT
TGGCATATTTTAG
